# Specifying wind gusts based on wind speed increments and forecasting gustiness

**DOI:** 10.1038/s41598-026-64301-5

**Published:** 2026-08-13

**Authors:** So-Kumneth Sim, Philipp Maass

**Affiliations:** Fachbereich Mathematik/Informatik/Physik, Universität Osnabrück, Barbarastraße 7, D-49076 Osnabrück, Germany

**Keywords:** Climate sciences, Engineering, Mathematics and computing

## Abstract

Wind gust forecasting is crucial for mitigating damage to people and property. We define gusts as rapid wind speed changes exceeding application-specific thresholds, and propose forecasting gustiness, that is, the number of gusts per time unit. For the forecasting, we employ a correlation between gustiness and variance of wind speed increments, quantified in an analysis of measured offshore data. By modeling speed increment variance with an autoregressive process, we construct a predictor for gustiness to surpass a threshold. The method is exemplified for rapid changes of wind-induced drag forces. After optimizing the forecasting procedure, we observe specificity comparable to a baseline persistence model, with significantly improved sensitivity. Our methodology of defining gusts and forecasting gustiness offers lots of room for improvements. Further developments may lead to high-quality forecasting in real-world applications.

## Introduction

Wind is a chaotic but ubiquitous phenomenon on Earth, with far-reaching consequences for both nature and technology. Its dynamics span many orders of magnitude^1^, from weather patterns persisting for several days, to sudden wind gusts on the scale of seconds. Meteorological forecasts are important for large-scale weather patterns on time scales of several hours or days^2,3^. For harvesting wind energy, forecasts on time scales of ten minutes are of interest^4^.

Less attention has been given so far to short-term forecasting of wind gusts^5–8^, despite their significant impact on several technical applications. A sparse Gaussian process regression model in combination with numerical weather prediction has been employed to improve the short-term and long-term wind gust forecasting^9^. For prediction of downburst winds, ARMA and GARCH time-series models have been developed^10^. Gust forecasting during a typhoon event was analyzed using a linear regression model and a micro-genetic algorithm^11^.

In aviation, unexpected wind gusts resulting from clear air turbulence cause vibrations and altitude changes, and can strongly affect the trajectory of an airplane^12,13^, leading to discomfort and possibly injuries^14,15^. Modern passenger aircraft often make use of so-called gust load alleviation systems^16–20^ to reduce such effects. For unmanned aerial vehicles of low weight, of growing interest in urban environments for delivery and other tasks, gusts can pose serious hazards near buildings^21,22^.

In power output from wind turbines, wind gusts cause large fluctuations, challenging the stability of power grids^23–26^. Forecasting sudden changes of electrical power, so-called power ramps, is needed for mitigating damage^27^ and ensuring stable operation of power grids^28–30^. With increasing feed-in of wind energy into power grids, ramps are expected to become more frequent^31^, and due to climate change, extreme wind speeds and frequencies of gusts are expected to increase^32–34^.

In outdoor sports competitions, strong winds can affect their execution. Studies of the Beijing 2022 Winter Olympic Games emphasize the importance of gust forecasting and in particular the difficulties in complex terrain^35,36^.

Despite their relevance as cause of damage and instabilities, there is no clear consensus yet on how gusts should be defined. In meteorology, a gust is often considered as the maximum wind speed observed within a specified measurement period^37–39^.

In load alleviation systems, gusts are defined as sinusoidal or cosine wind waves with specified wavelengths^40,41^. Modified cosine functions^42^ and other functions^43,44^ are considered also. For aircraft or buildings, it was argued that the spatial extent and duration of a gust must be large enough, which led to requirements of three to five seconds duration^45^. Shorter gust durations are of interest in view of increasing importance of smaller objects such as drones.

In Ref^46^., it was suggested that a gust is a sudden change of wind speed exceeding a constant threshold. We here follow this idea but refine the threshold criterion with respect to applications.

Since wind speed changes are rather weakly correlated^47^, it is not a good idea to attempt gust forecasting for individual time points. More appropriate is to forecast gustiness $\dot{N}$, defined as the number of gusts *N* per time unit in a certain time interval *τ*, $\dot{N}=N/\tau$. In particular, forecasting time intervals of high gustiness, exceeding a threshold $\dot{N}_\textrm{min}$, is of interest.

We will show that high gustiness is correlated with the variance of wind speed increments, and, surprisingly, only weakly with their kurtosis. In this study, we thus base forecasting of time intervals of high gustiness on local variances of wind speed increments in time windows of equal size.

**Fig. 1:**
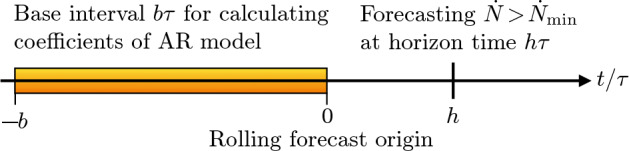
Forecasting procedure based on an AR process of local variances of wind speed increments: in a base time interval *bτ* preceding the rolling forecast origin, the coefficients of the AR process are calculated. At the forecast horizon *hτ*, it is forecasted whether gustiness $\dot{N}$ exceeds a threshold $\dot{N}_\textrm{min}$.

Various methods of time series analysis can be applied for predicting local variances of wind speed increments. We apply an autoregressive (AR) process. In a base time interval of length *bτ*, we determine the coefficients of the AR model. The end of the base time interval sets the forecast origin, see Fig. 1. To forecast whether gustiness $\dot{N}$ exceeds a threshold $\dot{N}_\textrm{min}$ after a time *hτ* from this origin, we apply the AR model. The time *hτ* hence defines the forecast horizon (or lead time) for threshold-exceeding gustiness $\dot{N}>\dot{N}_\textrm{min}$. The whole procedure is carried out with the forecast origin rolling.

We apply the method to offshore wind speed data sampled with a time resolution of one second over 20 months. As training period for optimizing model parameters, we use the first month. Evaluation of predictive power for time intervals of high gustiness in the subsequent 19 months shows that more than 90% of these intervals are correctly identified for most months. Among the intervals having $\dot{N}<\dot{N}_\textrm{min}$, between 3% and 32% are falsely forecasted to exhibit high gustiness.

## Wind gust definitions

Given the significant impact of sudden changes in wind speed on technical applications, this section provides precise definitions of gusts for different contexts. A general definition is based on the observation that rapid changes of wind speed *v* over a short time period *Δ t* can significantly affect aviation and wind energy. These changes are quantified by increments

1 $$\begin{aligned} u(t) = v(t+\Delta t) - v(t)\,, \end{aligned}$$

where increments are evaluated over time windows of length *Δ t*. Turbulence theory provides statistical insights into the properties of *u*^48–53^. Reference^46^ associates intermittency in turbulence with large increments and defines gusts as

2 $$\begin{aligned} u(t) \ge u_\textrm{th} \,. \end{aligned}$$

The choice of an appropriate threshold $u_\textrm{th}$ depends on the application. While a constant value is possible, it is often more realistic to consider a time-dependent threshold $u_\textrm{th}(t)$, as illustrated below for aviation and wind energy.

### Increment threshold in aviation

To make aviation more cost-effective, many aircraft are designed to be lightweight. The disadvantage of lighter aircraft is their lower inertia, which makes them more susceptible to the effects of wind gusts and to resulting hazards.

Sudden gusts impact aviation through changes in the drag force acting on an aircraft,

3 $$\begin{aligned} F = \frac{c_\textrm{D}}{2} \rho A v^2\,, \end{aligned}$$

where *ρ* is air density, *A* is the effective cross-sectional area perpendicular to the flow, and $c_\textrm{D}$ the drag coefficient. For aircraft in motion, *v  *denotes the true airspeed relative to the surrounding air. In Ref^15^. gusts were defined according to their acceleration of aircraft.

Rapid changes in *v* imply rapid changes in *F*, and thus in the acceleration $\dot{v}$ according to Newton’s law $F = m \dot{v}$, with *m* the aircraft mass. The time derivative of the acceleration, the jerk $\ddot{v}$, is hazardous when it exceeds a critical value $\ddot{v}_\textrm{th}$^54,55^. Approximating the time derivative of *F* by a difference quotient yields

4 $$\begin{aligned} \ddot{v}(t) \simeq \frac{c_\textrm{D}}{2} \frac{\rho A}{m} \frac{v^2(t+\Delta t)-v^2(t)}{\Delta t}\,, \end{aligned}$$

where *Δ t* corresponds to a typical response time of the propulsion system. Setting $\ddot{v}(t) \le \ddot{v}_\textrm{th}$ defines a gust threshold,

5 $$\begin{aligned} u_\textrm{th}(t) = \left[ \frac{2m}{c_\textrm{D}\rho A} \Delta t\hspace{0.7pt}\ddot{v}_\textrm{th} + v^2(t) \right] ^{1/2} - v(t)\,. \end{aligned}$$

An alternative definition is based on relative drag changes, requiring

6 $$\begin{aligned} u_\textrm{th}(t) = \left( \sqrt{1+F_\textrm{th}^\textrm{rel}} - 1 \right) v(t)\,, \end{aligned}$$

where $F_\textrm{th}^\textrm{rel}$ is a relative drag threshold. This definition is independent of *ρ*, *m*, and *A*, but Eq. (5) is more suitable when aircraft-specific parameters are relevant.

### Increment threshold in wind energy harvesting

Power ramps lead to frequency instabilities in power grids^31,56,57^. They are commonly defined as changes exceeding a threshold in some time interval. Other definitions regard power ramps as events where the power output of the wind farm changes by a given percentage of the total installed capacity^58,59^. Responses of power frequencies to wind fluctuations can be fast. They occur on time scales even smaller than one second^60^.

The power output of a turbine with rotor radius *R* is

7 $$\begin{aligned} P = \frac{c_\textrm{P}}{2} \pi R^2 \rho v^3\,, \end{aligned}$$

where $c_\textrm{P} \le 16/27$ according to Betz’s law^61,62^. A gust can be defined as an increment *u*(*t*) that causes a power increase *P(t+Δ t)-P(t)* above a threshold $P_\textrm{th}$, which yields

8 $$\begin{aligned} u_\textrm{th}(t) = \left[ \frac{2}{c_\textrm{P} \pi R^2 \rho } P_\textrm{th} + v^3(t) \right] ^{1/3} - v(t)\,. \end{aligned}$$

Alternatively, a relative threshold can be used by setting $P_\textrm{th} = c_\textrm{th} P_\textrm{max}$, where $P_\textrm{max}$ is the rated capacity and $c_\textrm{th}$ a critical capacity factor.

Real turbines deviate from the ideal cubic law: below cut-in speed no power is generated, between cut-in and rated speed the cubic law holds approximately, above rated speed power output saturates, and beyond cut-out speed turbines shut down^63–65^. Thus, Eq. (8) must be adapted to the actual turbine power curve.

Given the mechanical inertia of the rotor blades of a wind turbine and the electric generator, one may wonder whether wind gusts on time scales of seconds play a role for the power feed-in into electricity grids. Remarkably, due to the strong non-Gaussian features of the statistics of wind speed increments, rapid changes of wind power on scales of seconds are transferred to electrical power^24,60,66,67^.

In summary, gusts are regarded as strong wind speed changes over a time period *Δ t*, corresponding to increments *u*. These increments are identified as gusts if they exceed a threshold $u_\textrm{th}(t)$, which may be constant or time-dependent depending on the application.

## Gusts in offshore wind speed data

To investigate how gusts show up and how to define suitable quantities for forecasting, we use high-resolution offshore wind speed measurements obtained at the FINO1 research platform.

**Fig. 2:**
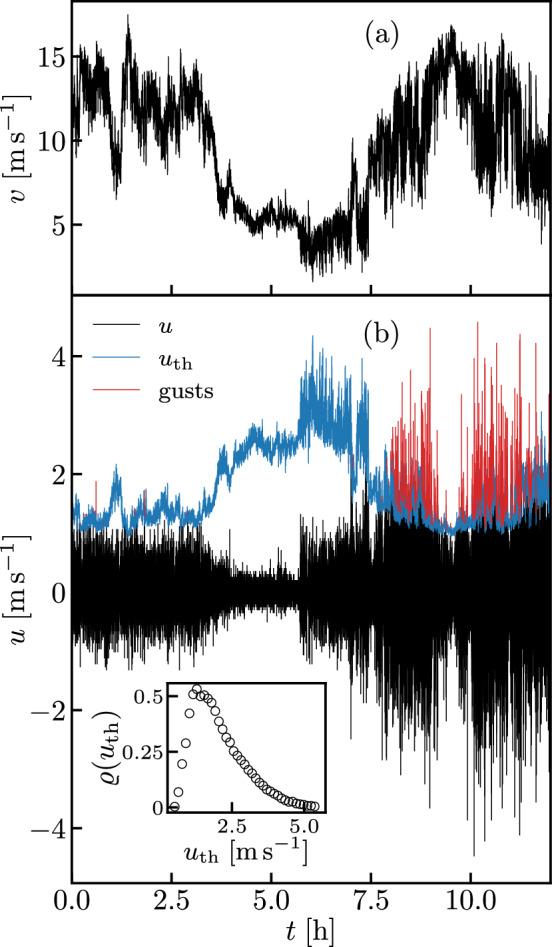
Twelve-hour segment (**a**) of wind speeds *v* with resolution $\Delta t=1\,{\textrm{s}}$ and (**b**) of their corresponding increments *u*. The blue line in (**b**) shows jerk-based gust thresholds $u_\textrm{th}$ [Eq. (5)] for $\ddot{v}_\textrm{th}=2.1\,{\hbox {m}\,\hbox {s}^{-3}}$, $c_\textrm{D}=0.1$, $A=1\,{\hbox {m}^2}$, $\rho =1.2\,{\hbox {kg}\, \hbox {m}^{-3}}$ and $m=1\,{\hbox {kg}}$. Occurring gusts are marked in red. The distribution of $u_\textrm{th}$ over the entire time series is shown in the inset of (**b**).

**Fig. 3:**
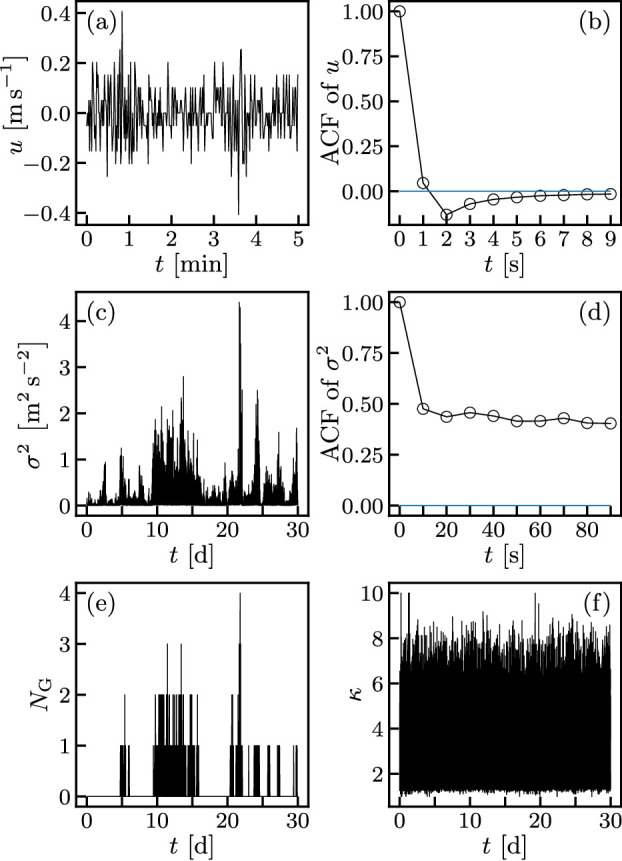
Gustiness and volatility of wind speed increments. (**a**) Representative example of wind speed increments *u*(*t*) over a time period of $5\,\textrm{min}$ and (**b**) ACF of increments *u*. (**c**) Local variance $\sigma ^2$ in time windows $\tau =10\,{\hbox {s}}$ and (**d**) ACF of this local variance. (**e**) Number $N_{\scriptscriptstyle G}$ of gusts and (**f**) local kurtosis *κ* in time windows of length *τ*. Gusts are specified according to the jerk-based definition in Eq. (5) with the same parameters as in Fig. 2.

FINO1 is located in the North Sea about $45\,{\hbox {km}}$ north of the island of Borkum. The mast extends up to $100\,{\hbox {m}}$ above sea level and is equipped with three-cup anemometers at eight different heights between $30\,{\hbox {m}}$ and $100\,{\hbox {m}}$. Each anemometer records the magnitude $(v_x^2+v_y^2)^{1/2}$ of the horizontal wind velocity, hereafter simply referred to as wind speed *v*(*t*). The dataset used in this study consists of measurements with a temporal resolution of $\Delta t=1\,{\hbox {s}}$ collected over 20 months from September 2015 to April 2017 at a measurement height of $90\,{\hbox {m}}$. Occasional gaps occur due to sensor malfunctions such as icing, but the overall fraction of missing data is below 1%. When calculating averaged quantities, these missing entries are omitted. We analyze increments *u*(*t*) with lag $\Delta t=1\,{\hbox {s}}$, as defined in Eq. (1).

**Fig. 4:**
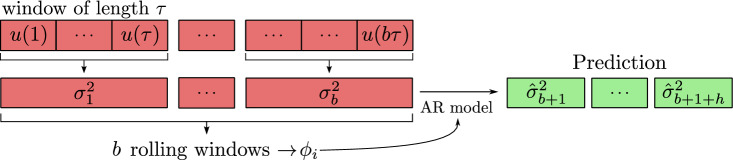
Illustration of the AR-based prediction of local variances of wind speed increments. The upper array shows the time series of increments *u* with resolution $\Delta t=1\,\textrm{s}$, and the lower array the series of their local variance in non-overlapping time windows of length *τ*. A sequence of *b* consecutive variances defines the base interval of length *bτ*, used for estimating the AR parameters. Predicted variances $\hat{\sigma }^2_{b+1+j}$, *j=0,… , h*, are marked in green. For *h=0*, only $\hat{\sigma }^2_{b+1}$ is predicted.

Figure 2 illustrates the jerk-based gust definition (5) on a twelve-hour segment of the time series with resolution $\Delta t=1\,{\hbox {s}}$ for a set of parameters $\ddot{v}_\textrm{th}$, $c_\textrm{D}$, *A*, *ρ* and *m* given in the figure caption. Panel (a) shows alternating time periods of high and low wind speed *v*, and panel (b) the corresponding increments *u*. The blue curve depicts the time-dependent jerk-based threshold $u_\textrm{th}$ from Eq. (5). Because $\textrm{d}u_\textrm{th}/\textrm{d}v<0$, $u_\textrm{th}$ is lower when *v* is higher.

### Gustiness and volatility of wind speed increments

To identify quantities suitable for forecasting, we first analyze statistical features of wind speed increments. Figure 3a shows *u*(*t*) for a $5\,{\hbox {min}}$ time interval, and Fig. 3b displays the autocorrelation function (ACF) of the full time series *u*(*t*). This ACF shows only very weak anticorrelations for time lags 1–5 s. We thus do not attempt to forecast gusts at specific time instants based on the correlation properties of *u*(*t*).

A more suitable approach is to forecast whether the number of gusts in a time window of length *τ* exceeds a minimal number $N_\textrm{min}$. We define the total number $\dot{N}$ of gusts per time unit as gustiness, $\dot{N}=N/\tau$, and aim to forecast time intervals of high gustiness, where $\dot{N}>\dot{N}_\textrm{min}$. Such time intervals are identified by the binary indicator

9 $$\begin{aligned} G_j=\left\{ \begin{array}{ll} 1\,, & \dot{N}_j>\dot{N}_\textrm{min}\,,\\ 0\,, & \textrm{otherwise}\,. \end{array}\right. \end{aligned}$$

For the forecasting, it is possible to consider prediction methods for the time series $\dot{N}_j$. However, as $\dot{N}_j$ is a discrete variable for given *τ*, well-established methods for continuous variables cannot be applied. Methods for forecasting ordinal time series are less established, although research is currently ongoing to develop suitable, tailor-made procedures^68,69^.

Alternatively, one can search for continuous variables strongly correlating with $\dot{N}$. Measures quantifying the volatility of *u*(*t*) are good candidates for such variables, for example, the variance of *u*(*t*) in time windows of length *τ*.

Specifically, we consider non-overlapping intervals $[1+(j-1)\tau ,j\tau ]$, *j=1,2,…*, of the series *u*(*t*), see Fig. 4. For each interval *j*, the variance $\sigma ^2_j$ of the increments *u*(*t*) and the gustiness $\dot{N}_j=N_j/\tau$ are calculated. For the window size *τ*, we choose $\tau =10\,{\hbox {s}}$. Longer *τ* can be taken if this is more appropriate for a specific application.

A quantitative analysis yields a Pearson correlation coefficient 0.75 between $\sigma ^2_j$ and $\dot{N}_j$. This correlation is evident also from Figs. 3c and e, where we show $\sigma ^2_j$ and $\dot{N}_j$ for 30 days. Remarkably, the kurtosis $\kappa _j$ of *u*(*t*) in the non-overlapping windows does not strongly correlate with $\dot{N}_j$, see Fig. 3f.

Contrary to the ACF of *u*(*t*), the ACF of the local variances $\sigma ^2_j$ on scale *τ* shows strong and persistent correlations over long time lags up to several hours ($10^4\,{\hbox {s}}$), see Fig. 3d.

Hence, it is tempting to take a threshold $\sigma ^2_\textrm{th}$ for the local variance as a predictor for intervals of high gustiness $\dot{N}>\dot{N}_\textrm{min}$, corresponding to the binary predictor

10 $$\begin{aligned} \hat{G}_{k+1+h}=\left\{ \begin{array}{ll} 1\,, & \hat{\sigma }^2_{k+1+h}>\sigma ^2_\textrm{th}\,,\\ 0\,, & \textrm{otherwise}\,. \end{array}\right. \end{aligned}$$

Here *k* is the index of the last past time interval *τ* of known wind speed data, and *h* the index of the forecast horizon interval, with *h=0* corresponding to the interval directly following the last interval of known speeds.

An evaluation of the FINO data for successive months indeed shows that the predictor $\hat{G}$ has fairly good quality. In this evaluation, we assume that prediction of the local variances is perfect, i.e. given by an oracle model yielding $\hat{\sigma }^2_j=\sigma _j^2$. Errors in the forecasting of high gustiness intervals in the oracle model are solely due to the imperfect correlation between $\dot{N}$ and $\sigma ^2$. An optimized $\sigma _\textrm{th}^2$ for the perfect predictor of the variances is searched for, yielding a high sensitivity and high specificity for the gustiness prediction. The sensitivity is the probability of obtaining $\hat{G}_j=1$ if $G_j=1$, and the specificity is the probability of finding $\hat{G}_j=0$ if $G_j=0$. The sensitivity is also called true positive rate (TPR). One minus the specificity gives the false positive rate (FPR). Specifically, we determine the optimized $\sigma _\textrm{th}^2$ by maximizing Youden’s index^70^

11 $$\begin{aligned} J = \textrm{TPR} - \textrm{FPR}\,. \end{aligned}$$

The optimized values in the successive months of the FINO data give high TPR above 93% and fairly low FPR in the range 1–19% for the oracle model (see also later the corresponding data in Fig. 6).

In conclusion, our analysis reveals that intervals of high gustiness can be forecasted by predicting local variances $\sigma _j^2$ of wind speed increments. In the following, we consider as a standard approach a simple autoregressive (AR) model for $\sigma ^2_j$ and evaluate the predictive power when using this model.

## AR model for local variance of wind speed increments

An AR process of order *p* for predicting $\hat{\sigma }^2_{j+1}$ from knowledge of *p* previous local variances is given by

12 $$\begin{aligned} \hat{\sigma }^2_{j+1}=\phi _0 +\sum _{i=1}^{p} \phi _i\, \sigma ^2_{j+1-i}\,, \end{aligned}$$

with coefficients $\phi _i$, *i=0,… ,p*, to be estimated. The AR process can be considered also as a modified autoregressive conditional heteroskedasticity (ARCH) model, where instead of the series $u^2(t)$ we use the $\sigma ^2_j$.

When assuming the AR process (12) to describe exactly the time evolution of the mean $\langle \sigma ^2\rangle$, $\phi _0$ must satisfy the relation

13 $$\begin{aligned} \phi _0=\Bigl (1-\sum _{i=1}^p\phi _i\Bigr )\langle \sigma ^2\rangle \,. \end{aligned}$$

Inserting this $\phi _0$ into Eq. (12) yields

14 $$\begin{aligned} \Delta \hat{\sigma }^2_{j+1}=\sum _{i=1}^{p} \phi _i\, \Delta \sigma ^2_{j+1-i}\,, \end{aligned}$$

where $\Delta \hat{\sigma }^2_j=\hat{\sigma }^2_j-\langle \sigma ^2\rangle$ and $\Delta \sigma ^2_j=\sigma ^2_j-\langle \sigma ^2\rangle$. Equation (14) is a standard AR process. Its coefficients follow when assuming the process to model exactly the evolution of the correlations $C_j=\langle \Delta \sigma ^2_i\Delta \sigma ^2_{i+j}\rangle =C_{-j}$. The determining equations for the $\phi _i$ then are the Yule-Walker equations^71,72^

15 $$\begin{aligned} \begin{pmatrix} C_0 & C_{-1} & \cdots & C_{-p+1} \\ C_1 & C_0 & \cdots & C_{-p+2} \\ \vdots & \vdots & \ddots & \vdots \\ C_{p-1} & C_{p-2} & \cdots & C_0 \end{pmatrix} \begin{pmatrix} \phi _1 \\ \phi _2 \\ \vdots \\ \phi _p \end{pmatrix} = \begin{pmatrix} C_1 \\ C_2 \\ \vdots \\ C_p \end{pmatrix}\,. \end{aligned}$$

For predicting $\hat{\sigma }^2_{j+1}$, we determine coefficients $\phi _i$ by using a number *b* of known successive values $\sigma ^2_j,\sigma ^2_{j-1}\ldots ,\sigma ^2_{j-b+1}$, see Fig. 4, and determine for this window the mean $\langle \sigma ^2\rangle$ and the correlations $C_j$. Inserting these $C_j$ into Eq. (15) and solving the system of linear equations yields the $\phi _1,\ldots ,\phi _p$. Inserting $\langle \sigma ^2\rangle$ and the $\phi _i$ in Eq. (13) gives $\phi _0$.

The procedure is carried out with a rolling base time interval by continuous measurement of the wind speeds. For the next predictor $\hat{\sigma }^2_{j+2}$, $\sigma ^2_{j+1}$ is updated from the continuous measurements and the $\phi _i$ are determined from the *b* successive values $\sigma ^2_{j+1},\sigma ^2_j\ldots ,\sigma ^2_{j-b}$.

The above description is for the forecast horizon *h=0*, i.e. $\hat{\sigma }^2_{j+1}$ is predicted from the forecast origin *j*. For larger forecast horizons *h>0*, the coefficients $\phi _i$ from the same training window are used but the unknown values $\sigma ^2_{j+1},\ldots ,\sigma ^2_{j+h-1}$ on the right hand side of Eq. (12) are replaced by the predicted values $\hat{\sigma }^2_{j+1},\ldots ,\hat{\sigma }^2_{j+h-1}$. For *h\ge p*, in particular, solely predicted values for the variances enter the calculation of $\hat{\sigma }^2_{j+h}$ in Eq. (12).

### Parameter optimization

In an application with given gust specification, e.g. the jerk-based one in Eq. (5), it is of interest when the gustiness exceeds a critical threshold in a future time window, i.e., the quantities $\dot{N}_\textrm{min}$, *τ*, and *h* are set. As an example, we take here the same $\tau =10\,{\hbox {s}}$ as for the analysis in Fig. 3, $\dot{N}_\textrm{min}=0.2\,\textrm{s}^{-1}$ corresponding to the dashed line in Fig. 3e, and *h=6*, corresponding to forecasting high gustiness in a time interval $[60\,{\hbox {s}},70\,{\hbox {s}})$ ahead of the forecast origin.

The method needs to be optimized with respect to the parameters *p* and *b* entering the rolling AR process, and with respect to the threshold $\sigma ^2_\textrm{th}$ used for guessing high gustiness intervals.

A best set of parameters *p*, *b*, and $\sigma ^2_\textrm{th}$ is searched for maximizing Youden’s index in Eq. (11). As training period, we take the first month of the FINO time series (September 2015).

**Fig. 5:**
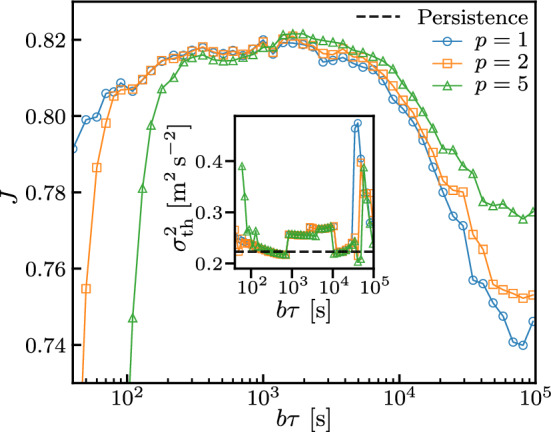
Variation of Youden’s index *J* with the base time interval *bτ* for three fixed values of the order *p* of the AR process in Eq. ((12)) and optimized thresholds $\sigma ^2_\textrm{th}$ of the variance of wind speed increments. These variances serve as predictors of high gustiness in Eq. ((10)) and are displayed in the inset. The time window for determining gustiness $\dot{N}$ is $\tau =10\,{\hbox {s}}$. Intervals of high gustiness are when $\dot{N}$ exceeds $\dot{N}_\textrm{min}=0.2\,\textrm{s}^{-1}$. The forecast horizon is *h=6*, corresponding to forecasting high gustiness in a time interval $[60\,{\hbox {s}},70\,{\hbox {s}})$.

Figure 5 shows *J* as a function of the base time interval *bτ* for three fixed values of *p* and the best $\sigma ^2_\textrm{th}$ for given *p* and *b*, i.e. the one yielding the highest *J* value. These best $\sigma ^2_\textrm{th}$ values are plotted in the inset of the figure. Remarkably, increasing the order *p* beyond one has almost no effect on *J*. We hence choose the smallest value *p=1*. The largest *J* then is obtained for the optimal parameters $b\cong 2\times 10^2$ ($b\tau \cong 2\times 10^3\,{\hbox {s}}$) and $\sigma ^2_\textrm{th}\cong 0.25\,{\hbox {m}^2\hbox {s}^{-2}}$.

We have checked that comparable results are obtained when choosing other months for the optimization of the parameters *p*, *b* and $\sigma ^2_\textrm{th}$, i.e. the calibration of the model is not strongly affected by seasonal effects. For all months, the dependence of the Youden’s index on the order *p* of the AR model is very weak, the optimal value of the base time interval *bτ* lies between $10^3\,\textrm{s}$ and $3\times 10^3\,\textrm{s}$, and the optimal threshold value $\sigma ^2_\textrm{th}$ fluctuates in the range $0.2-0.3\,\textrm{m}^2\textrm{s}^{-2}$.

### Results

We apply the forecasting procedure based on the AR model with optimized parameters from the first month to the remaining 19 months of the FINO wind speed data.

**Fig. 6:**
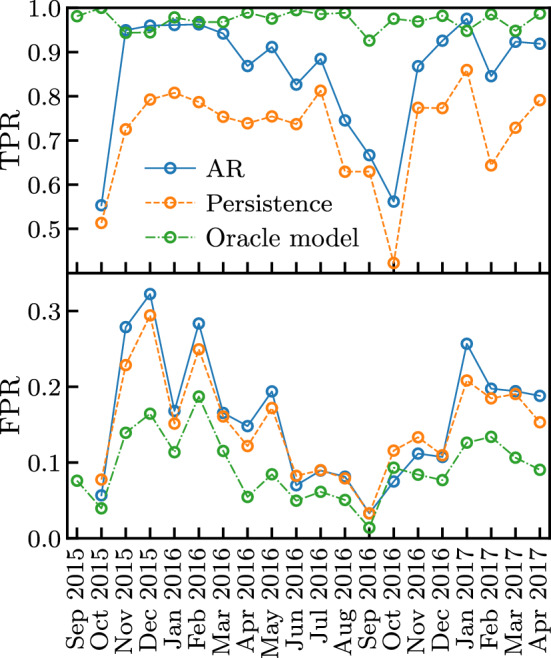
Monthly variation of TPR and FPR for forecasting intervals of high gustiness based on predicting local variances of wind speed increments by the AR model with order *p=1* and based on simple variance persistence as baseline model for comparison. The parameters of the AR model are obtained by using the first month (September 2015) as training period with known wind speed data. Results of the oracle model for intervals of high gustiness were determined by assuming perfect prediction of the local variances for each month.

For evaluation of the forecasting, we compare our results to two baseline models. The first is a simple persistence model, where the predicted variance $\hat{\sigma }^2_{j+h}$ is $\sigma _j^2$, i.e. given by the last known value. The second is an oracle model with respect to the local variances of wind speed increments, that means it is assumed that the $\hat{\sigma }_j^2$ are exactly predicted, $\hat{\sigma }_j^2=\sigma _j^2$. In this model, errors in the forecasting of high gustiness intervals originate solely from the imperfect correlation between $\dot{N}$ and $\sigma ^2$.

Figure 6 shows the quality of the forecasting, quantified by the TPR and FPR in successive months. The TPR varies in the range 54–97% with an average of 86%. It exhibits similar variations to those of the persistence model. The FPR lies in the range 3–32% with an average of 16%, and it varies in a similar way as both baseline models. As expected from the general tradeoff between sensitivity and specificity, a high TPR in most cases goes along with a high FPR and vice versa. In months with few intervals of high gustiness, most notably August–October 2016, the fixed threshold $\sigma ^2_\textrm{th}$ optimized on the training month is less well matched to the lower variance, so that the AR forecasts rarely exceed it and the TPR drops. This affects the AR and persistence models but not the oracle one, where the threshold is reoptimized for each month.

Compared to the persistence model, the TPR is significantly improved, whereas this is not the case for the FPR. The oracle model yields a TPR of 97% (range 93–99%) and a mean FPR of 10% (range 1–19%). The comparatively high FPR even for perfect prediction of the increment variances shows that the forecasting based on the correlation between $\dot{N}$ and $\sigma ^2$ has principal limits. The data for the oracle model furthermore demonstrate that both TPR and FPR can be improved by better predictors for the variances than provided by the AR model.

We have checked that our findings are robust when increasing the time interval *Δ t* in Eq. (1) for the wind speed increments, when keeping all other parameters fixed. For $\Delta t=2\,\textrm{s}$, the calibration yields essentially the same behavior as reported above for $\Delta t=1\,\textrm{s}$: Youden’s index *J* is maximal for a base time interval *bτ* between $10^3\,\textrm{s}$ and $3\times 10^3\,\textrm{s}$, and increasing the order *p* does not lead to higher *J*. The optimal threshold $\sigma ^2_\textrm{th}$ becomes larger by a factor of about three. The monthly evaluation for $\Delta t=2\,\textrm{s}$ in terms of TPR and FPR shows almost the same behavior as in Fig. 6.

We also have obtained comparative results when varying, at fixed other parameters, the time window *τ* for determining the gustiness $\dot{N}=N/\tau$. When decreasing *τ* to 5 s, the overall behavior of the TPR and FPR is similar. The TPR values for $\tau =5\,{\hbox {s}}$ and 10 s are also similar, while the FPR for $\tau =5\,{\hbox {s}}$ is larger by a factor of about 1.5. The reason for this higher FPR is that the correlation between the local variance $\sigma ^2$ of the wind speed increments and the gustiness $\dot{N}$ becomes weaker for smaller *τ*: it decreases from 0.75 at $\tau =10\,\textrm{s}$ to 0.67 at $\tau =5\,\textrm{s}$.

## Conclusions

We have defined wind gusts as changes *u(t)=v(t+Δ t)-v(t)* of wind speeds in a time interval *Δ t* if they exceed a threshold $u_\textrm{th}$. While the threshold can be taken as constant, it should be more appropriate to use time-varying $u_\textrm{th}(t)$ in relevant applications, as we exemplified by considering wind-induced jerks in aviation and power ramps in wind energy harvesting.

By analyzing offshore wind speed data, we showed that forecasting such wind gusts at individual time points is hampered by the weak and short-ranged anticorrelations between wind speed increments. We therefore suggest to focus on forecasting time intervals *τ* with a critically high number *N* of gusts larger than a threshold, or, differently speaking, with a high gustiness $\dot{N}=N/\tau$ exceeding a rate $\dot{N}_\textrm{min}$.

To study the potential of this approach, we have based our forecasting on the correlation between gustiness and local variance of wind speed increments. An analysis of the measured wind speed data revealed a Pearson correlation coefficient of 0.75 between the time series $\sigma ^2_j$ of local increment variance and the series $\dot{N}_j$ of gustiness, and the series $\sigma _j^2$ showed pronounced long-range correlations. These findings suggest that it is possible to develop good predictor models for the local variance and that forecasting intervals *j* with $\dot{N}_j>\dot{N}_\textrm{min}$ can be related to predicting $\sigma _j^2$ exceeding a threshold $\sigma ^2_\textrm{th}$.

For predicting $\sigma _j^2$, we have applied a rolling AR model. Its parameters and the threshold $\sigma ^2_\textrm{th}$ were optimized by maximizing the difference (Youden’s index) TPR-FPR between true and false positive rates for forecasting $\dot{N}_j>\dot{N}_\textrm{min}$. This optimization was carried out for an example set of application parameters and by taking the first month of measured wind speeds as known training data. The remaining 19 months of the data were used to evaluate the forecasting.

Comparisons of the results with a persistence baseline show a significantly improved TPR at comparable FPR. As for the FPR, we found it to vary between 1–19% even under the assumption of perfect prediction of local variances of wind speed increments. This shows that forecasting of high gustiness intervals based on local variances of wind speed increments has principal limits.

Having considered a rather simple forecasting model here, there is room for improvement. Rather than using a linear regression model for predicting local variances of wind speed increments, more advanced nonlinear modeling techniques can be applied^73^. Nonlinear relations can be searched for to better quantify the relation between gustiness and the local variance. Statistical measures correlating with the gustiness, in addition to the local variances, can be taken into account in the forecasting procedure. For modeling gustiness $\dot{N}$ directly, it is possible to use autoregressive or count models for series of discrete variables^68,69^. Thereby, principal limits are overcome that arise when correlating gustiness with other statistical measures. Finally, one may employ machine learning algorithms for modeling gustiness^35,36,74–78^.

In view of these promising options, we believe that our basic approach of gust specification and gustiness forecasting can be improved to a level that makes it useful for real-world applications in the future.

## Data Availability

The authors declare that all data supporting the findings of this study are available within the paper or available from the corresponding author upon request.
